# A Pilot Tool of the Virtual Scenario Initial Dementia Cognitive Screening (VSIDCS) with a Cultural Exhibition for Improving the Standard Traditional Test

**DOI:** 10.3390/healthcare9091160

**Published:** 2021-09-04

**Authors:** Cheng-Li Liu, Shin-Ray Chang

**Affiliations:** Department of Industrial Management, Vanung University, Taoyuan 320313, Taiwan; srchang@mail.vnu.edu.tw

**Keywords:** dementia, episodic memory, mild cognitive impairment, virtual reality, involvement, emotion

## Abstract

Dementia has become a serious global health problem for older people. In the past, primary screening for dementia was carried out by a paper test. These standard traditional tests (e.g., Montreal Cognitive Assessment (MoCA) and Mini-Mental State Examination (MMSE)) have been used for many years. In addition to paper tests, is there another way to let people have better involvement and emotions during the test procedure? With the advancement of technology, the application of virtual reality (VR) and augmented reality (AR) have changed and improved many medical technologies. However, there are few applications of VR and AR in dementia screening. The purpose of this study was to apply VR and AR to construct a pilot tool for virtual scenario initial dementia cognitive screening (VSIDCS) with a cultural exhibition, to achieve better involvement and emotions in participants. There were three operating interfaces designed for the system: a VR screening interface, cognitive board, and AR recognition interface. There were twenty-four middle-aged people (Female 10 and Male 14 between 50 and 65 years of age and with an average age of 58.7 years) selected for the test. The results of the experiments showed that VSIDCS test scores are consistent with those of the MoCA and MMSE. Additionally, VSIDCS can induce better involvement and emotions than the MoCA and MMSE. Participants showed better enthusiasm and more positive experiences during the VSIDCS test.

## 1. Introduction

In recent years, due to the increase in average life expectancy, the incidence of age-related diseases has increased dramatically. The most common age-related symptom is mild cognitive impairment (MCI), which usually represents the transition from healthy aging to dementia. Dementia is a neurodegenerative disease that currently cannot be cured by medicine. According to the June 2020 monthly report of the Taiwan Dementia Association, there were 292,102 people with dementia in Taiwan at the end of 2019, of which 280,783 were over 65 with dementia, and 11,319 people with dementia between 45–64 years old. The overall dementia population approached 1.24% of the national population. It was estimated that by 2165, Taiwan’s dementia population will approach 900,000 [1]. According to a report by the World Health Organization, the global annual expenditure on dementia is over 604 billion US dollars [2]. In high-income countries, 45% of dementia expenditures are used for informal care, 40% are used for formal social care, and direct medical expenditures account for only 15%. Some normal aging phenomena (such as slow movement, memory loss, etc.) may be the initial signs of dementia in older people [3,4]. According to past research, MCI is an important indicator for early detection of dementia. For the determination of MCI, cognitive screening is an important first step. Screening for initial potential signs can be used for early diagnosis [3,5]. Subsequently, people with MCI can participate in prevention programs in advance to delay deterioration [6,7].

### 1.1. Early Signs of Dementia

Among the many aging phenomena, memory decline of varying degrees is the most commonly observed sign of dementia. Regardless of whether it is a healthy older adult or a person with a neurological disease, their earliest memory deterioration is usually an episodic memory deficit [8]. Due to the high correlation between perception and episodic memory, the decline in perception ability due to aging directly reduces the ability to encode and retrieve episodic memories. Episodic memory is a neurocognitive (brain/mind) system, uniquely different from other memory systems, that enables human beings to remember past experiences; it is usually a kind of integrated memory. In addition to the important components of an event, the contents of episodic memory also include the time, background environment, and related information accompanying the event. If there is a defect in multiple perceptions, it will lead to more serious episodic memory impairment. Some researchers claim that episodic memory contains the widest range of memories. When brain function begins to degrade, episodic memory is the first system impacted [9,10]. According to studies of brain injury cases, the hippocampal gyrus and prefrontal cortex play an important role in this complex memory system [11]. However, many researchers found that the neural circuits required for episodic memory can be separated from those of other learning and memory capabilities; the onset of aging in these neural circuits may be earlier or the degree of degeneration may be particularly serious, resulting in substantial degradation [12,13,14].

In addition, there is a blurred line between full cognitive functioning and dementia. Epidemiological studies have shown that MCI may be the prodromal stage of dementia [15,16]. MCI is a neurological disorder that mainly occurs in middle-aged individuals and older individuals. Main features include easily forgetting recent events, interruption of thoughts or speech, and forgetting words, appointments, or some special days. In addition to memory, other cognitive functions such as making decisions, estimating time, navigating complex tasks, and dealing with complex financial problems, planning, or organizational skills are affected, but daily life, work, and social interaction are not. At least one-third of people with MCI will develop dementia within 5 years. Those who are diagnosed with MCI and actively seek medical treatment at outpatient clinics will have a 40–50% rate of developing dementia within 5 years. When cognitive function continues to deteriorate, the person with MCI begins to experience independent living, social, and work difficulties, or mental disease symptoms (such as irritability and personality changes). These are symptoms of early (mild) dementia. In addition to memory problems, dementia can also be a manifestation of cognitive and behavioral disorders other than memory disorders [17]. Normal aging has little effect on daily life and brings only some inconveniences, but dementia greatly affects daily life and prevents individuals from taking care of themselves. Dementia is a disease that progresses slowly and is currently incurable. Early detection and treatment can effectively delay deterioration and reduce the pressure on caregivers to maintain the function and quality of life of people with MCI.

### 1.2. Initial Evaluation of Dementia

There are some tools used to screen dementia. The most commonly used tools are as follows:

1. Mini-Mental State Examination (MMSE)

The MMSE is a cognitive function assessment form designed by Folstein et al. in 1975 [18]. It is one of the most commonly used dementia assessment tools. Evaluation items include orientation (time and place), language (reading, writing, naming, understanding), constructiveness (visual drawing), attention and calculation ability (message registration and series minus 7), memory (short-term memory), and other functions. There is no time limit for the evaluation process, and the maximum score is 30 points. A score of 24 is the international standard cutoff value, a score of 18 to 24 indicates mild dementia, a score of 16 or 17 indicates moderate dementia, and a score of ≤15 indicates severe dementia. Due to different education levels, the cutoff values are also different. For subjects whose education levels are 7th grade or lower, the cutoff score on the MMSE is 22; for 8th grade or some high school (but not a graduate of), the cutoff score on the MMSE is 24; for high school graduates, the cutoff score on the MMSE is 25; and for some college or higher, the cutoff score on the MMSE is 26 or below. Although the MMSE is simple and easy to administer, it includes no scale for detecting emotions, personality, or behavior [19].

2. Montreal Cognitive Assessment Test (MoCA)

The MoCA was formulated by Nasreddine (2005) [20] in Canada based on clinical experience and cognitive behaviors. The test form is a scale used to quickly screen cases of mild cognitive impairment and can assess cognitive functions in various areas, including concentration, execution, memory, language, visual space construction, abstract concepts, calculation, and positioning. The total assessment score is 30 points, and a score of 26 or more is typical. Previous researchers found that the MoCA is a useful tool for detecting early cognitive decline and dementia and can accurately screen and measure cognitive ability [21,22]. Related studies have also shown that the test has high reliability and internal consistency [7,20].

3. The Informant Questionnaire on Cognitive Decline in the Elderly (IQCODE)

The IQCODE is a set of cognitive function questionnaires for testing cognitive decline developed by Jorm et al. (1991) [23,24]. It asks the relatives of the subject to describe changes in the intelligence of the subject over the past 10 years to assess the decline in living ability [25]. There are many language versions of the questionnaire for elderly individuals, including a Taiwanese version. The greatest benefit of the questionnaire is that it is not affected by education level, sex, or previous occupation. Previous studies have shown that the IQCODE is indeed more suitable as a screening test than the MMSE for ethnic groups with very different levels of education [26].

4. Clinical Dementia Rating (CDR)

The CDR was designed by Hughes et al. in 1982 [27] to conduct phased assessments for senile dementia of the Alzheimer’s type (SDAT). Morris (1993) [28] developed the current clinical scale according to the evaluation rules. The scale is often used in clinical diagnosis to describe the dependence characteristics of dementia at different stages. Due to its overall assessment of the daily life and cognitive function of patients, CDR is one of the main tools for assessing the severity of dementia. The scale contains 6 functional items: memory, orientation, judgment and problem solving, community affairs, home and hobbies performance, and personal care. All items are divided into 5 functional levels: no dementia (CDR = 0), questionable dementia (CDR = 0.5), MCI (CDR = 1), moderate cognitive impairment (CDR = 2), and severe cognitive impairment (CDR = 3) (Khan, 2016). The scale has been experimentally confirmed to have a reliability of 83%. Morris (1993) [28] found that the CDR scale is highly correlated with other neuropsychological measures and helps increase the accuracy of assessment. Dementia is suspected if the score is greater than 0.5. The CDR has been translated into multiple languages, including Asian languages, and has proven to be highly reliable and valid [29].

One of the key points in the prevention of dementia is early detection. Delaying the age of onset of dementia can effectively reduce the total population with dementia. With the baby boom after the Second World War, the population between the ages of 50 and 60 has now reached its peak. This is the prime age range for early diagnosis of dementia or other cognitive impairments. If a person with dementia is detected early and undergoes a certain degree of systematic cognitive training, it will help delay the rate of degeneration [6]. In summary, the MMSE, MoCA, and IQCODE are assessments of cognitive impairment and useful tools for detecting early cognitive decline and dementia. Among them, the MMSE and MoCA are systemic response questionnaires that focus on objective cognitive thinking and responses to the current situation. The IQCODE is a recall response questionnaire that focuses on subjective cognitive thinking about past and current situations. Finally, the CDR is a structured questionnaire for those who already suffer from dementia, to understand its qualitative severity.

These traditional screening methods have been used for many years. In addition to paper tests, is there another way to let people have better involvement and emotions during participation in test procedures? With the advancement of technology, virtual reality (VR) and augmented reality (AR) are widely used in medical treatments. The application of VR and AR has changed and improved many medical technologies. However, there are few applications of VR and AR in dementia screening systems. Additionally, according to Murriello’s research (2015) [30], an exhibition with essential and relevant dialog could increase the interest of visitors. Therefore, the purpose of this study was to construct an initial dementia cognitive screening system within a virtual cultural exhibition. It was expected that exhibiting a living situation in VR and AR with culturally meaningful virtual guided tours could motivate and attract middle-aged people to participate in an initial dementia screening.

## 2. Materials and Methods

### 2.1. Development Pilot Tool of Virtual scenario Initial Dementia Cognitive Screening (VSIDCS)

#### 2.1.1. Background of VSIDCS

This study developed a pilot tool for virtual scenario initial dementia cognitive screening system (VSIDCS), operating on a computer to replace traditional paper tests. Regarding the description of past tools used in the clinical evaluation of dementia in the previous section, the IQCODE questionnaire is a subjective recall questionnaire which is not suitable for development on a computer, and the CDR is a diagnostic evaluation scale for those who are already living with dementia. Therefore, the MMSE and MoCA are the main reference frameworks for the development of VSIDCS. In addition, research reporting from the 2013 American Association for Neurological Diseases (ANA) annual meeting stated that the MoCA is superior to the MMSE in assessing slight differences in cognitive function in people with MCI [31]. In this report, according to the research of Helen Hochstetler, a pharmaceutical company in Indianapolis enrolled 555 subjects with varying degrees of cognitive impairment into the Alzheimer’s Disease Neuroimaging Initiative (ADNI) study. In people ranging from mild to full-blown dementia, those with MMSE scores near the upper end of its 30-point range showed a much broader spread of MoCA scores; this result shows that the MoCA is more effective in evaluating changes in cognitive function. Therefore, the screening system developed by this study will be primarily based on the MoCA but will also refer to some characteristics of the MMSE. In addition, this system coupled with the evaluation of unique flexible control and contextual memory characteristics of the virtual environment achieves higher interest for subjects and more accurate results.

#### 2.1.2. Architecture of VSIDCS

**Figure 1:**
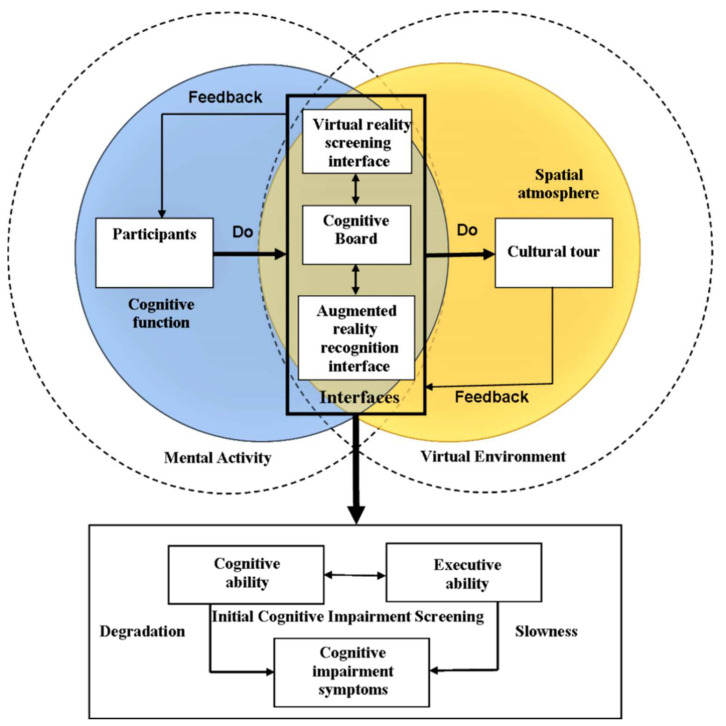
Architecture of the virtual scenario initial dementia cognitive screening (VSIDCS) system in a virtual cultural exhibition.

This study used the navigation concept of a virtual exhibition to guide participants to evaluate and screen for initial dementia. The screening system aimed to monitor the mental activities of participants during the navigation of a virtual cultural exhibition to generate comprehensive scores of cognitive and executive abilities (as shown in Figure 1). Individuals in the mood for a cultural visit could join the test. It was hoped that individuals would interact with cultural relics in the exhibition through the guided tour. Simultaneously, the individual’s cognitive function and episodic memory could be tested. Additionally, there was a health care worker from the retirement center in Taoyuan, Taiwan—whose jobs are general medical care and psychological counseling—that joined at the design stage to provide her opinions on matters including test items, scoring, and operating procedures. VSIDCS uses three operating interfaces to assess degradation and speed of participant cognitive and response abilities: a VR screening interface, a cognitive board, and an AR recognition interface.

**Figure 2:**
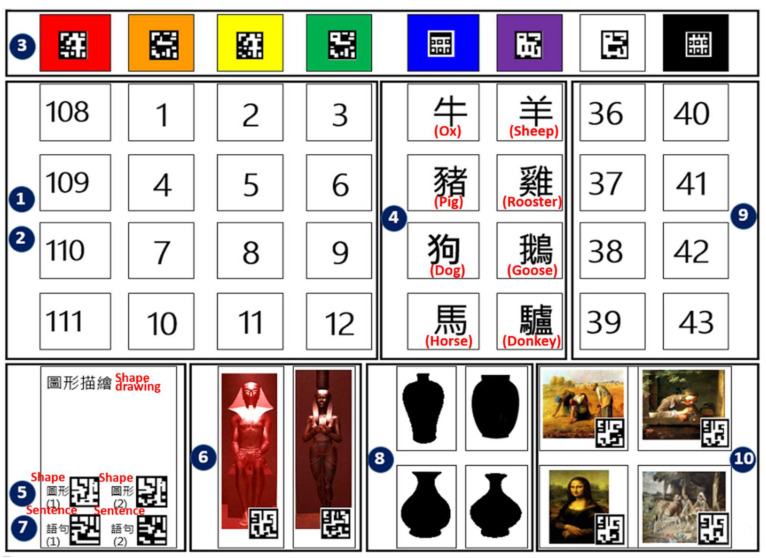
Cognitive board for the subject to answer questions.

1. VR screening interface: A virtual cultural relic exhibition was designed as a mental evaluation center. Participants enter this center as if they are visiting an exhibition. There were 4 exhibition areas designed in the center. Some specific testing questions were designed for each exhibition area; in this way, participants completed the test while visiting the exhibition.
2. Cognitive board: The board was an interface for participants to answer questions while visiting the virtual cultural exhibition (see Figure 2). Participants were asked to answer each question shown in the exhibition by scanning the answer’s code shown on the board with a mobile phone. The answer would be confirmed in the “AR recognition interface”, as explained in point 3.
3. AR recognition interface: The AR interface was designed for use on mobile phones. AR technology allows the virtual world on the screen to be combined and interact with real world scenes through positioning of the camera image and image analysis technology. Participants used a mobile phone to scan the answer’s code shown on the “cognitive board.” If the answer was correct, points were added.

#### 2.1.3. Characteristics of VSIDCS

VSIDCS is an original cognitive disability screening tool based on cultural relic guided tours. The cultural relic scenario is a virtual environment for cultural journeys. The purpose of VSIDCS was to conduct cognitive impairment assessments for middle-aged people (over 50 years old) through a virtual tour of cultural relics. This unique, dynamic, and stimulating cognitive assessment could establish a high-frequency (perhaps daily) mechanism for screening and monitoring the cognitive abilities of participants to improve the accuracy of cognitive screening for initial dementia. When a participant has signs of cognitive decline, in-depth diagnosis can be started immediately for a more comprehensive assessment. Therefore, VSIDCS plays a warning role for potential patients and medical experts. The characteristics of the system design are shown in Table 1.

#### 2.1.4. Scoring of VSIDCS

VSIDCS was primarily based on a standard traditional dementia test (i.e., MoCA), changing the pen and paper test into a computer test with a VR and AR environment. VSIDCS can automatically calculate total scores once completed to avoid manual calculation errors. However, some evaluation items such as vision/processing and language tests must be judged by the instructor. MoCA has obvious judgment difficulties in vision/processing and language tests; by comparison, MMSE is effective at these tests. For example, in the vision/processing test of the MoCA, the participant is asked to describe the structure of a clock and then manually judge whether the outline of a clock drawn by a participant is within a reasonable range. However, if the outline of the clock drawn by the participant is an ellipse, the system may have difficulty judging whether the shape is correct or wrong. Therefore, VSIDCS used the MMSE method of asking the subject to draw a specific pattern (such as a pentagon) for visual and process testing. In addition, in the oral comprehension test in the MoCA, the participant is asked to describe how oranges and bananas are similar. The correct answer is that both are fruit. However, it is also reasonable to say that both are peeled. Thus, there may not be a single correct answer, and a reasonable interpretation must be made by experts. Therefore, VSIDCS used the MMSE method of identifying specific artworks based on specific words for the participant to use in their responses to reduce the issue of multiple correct answers. Since both the MoCA and MMSE are administered via paper, it is not easy to evaluate the characteristics of dementia in control flexibility and episodic memory. VSIDCS used VR to design a unique situational environment that allows participants to complete the test during a cultural tour, without the test pressure that may be caused by paper questionnaires. Table 2 shows the evaluation items for cognitive assessment of initial dementia in the MoCA, MMSE, and VSIDCS. The corresponding points of the MoCA, MMSE, and VSIDCS are also described in the table.

#### 2.1.5. Situation Design of VSIDCS

This study was based on a cultural guide to constructing a virtual situation for cognitive screening of initial dementia. The exhibition environment in the VSIDCS was presented in the form of an arts exhibition hall so that participants could complete the test during a virtual tour. This could cause participants’ psychological pressure to be lower, and they would be more willing to take the test. According to the characteristics of the artworks, the exhibition hall was divided into four exhibition areas: lacquerware, pottery art, paintings, and statues. The objects were drawn with 3D MAX. The finished objects were edited for material and realistic design. Finally, an operating platform for the VSIDCS was built.

1. 1. Starting the test

**Figure 3:**
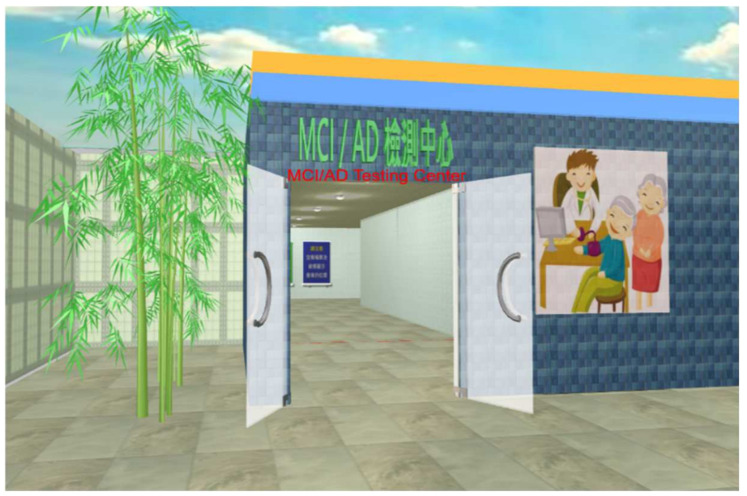
Startup screen of the VSIDCS.

When participants entered the operating screen of the VSIDCS (see Figure 3), they could use the keyboard or mouse to browse the exhibition. Each test was numbered. Participants could complete all tests in numerical order.

2. Orientation test

A. Time orientation

**Figure 4:**
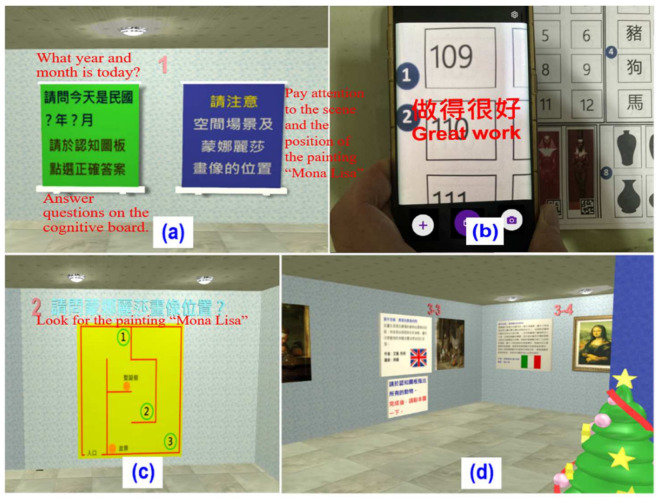
Orientation test screen: (**a**) Date questions in time orientation test; (**b**) Using mobile phone AR to answer date questions; (**c**) Instructions for looking for a specific painting “Mona Lisa”; (**d**) Location of the specific painting “Mona Lisa”.

Participants were asked to report the current date (year and month) during the orientation test, as shown in Figure 4a. Participants were asked to scan numbers on the “cognitive board” (see Figure 2) with a mobile phone to confirm answers. AR was used to respond to participants’ answers. The system gave feedback on the mobile phone “做的很好” (i.e., “great work”) whether they had scored correctly or not (see Figure 4b). If the answer was correct, points were added; the system did not add points if the answer was wrong.

B. Spatial orientation

Both the MoCA and MMSE have the disadvantage of not being able to test spatial orientation. To compensate for this shortcoming, the VSIDCS applied spatial operations in VR to assess spatial orientation. When a participant completed the “time orientation” assessment, she/he was asked to look for a specific painting (see Figure 4c). If the participant found the painting (see Figure 4d), she/he was asked to scan the identification number code on the “cognitive board” with a mobile phone. The system also gave feedback on the mobile phone “做的很好” (i.e., “great work”) whether they had scored correctly or not.

3. Memory test

A. Short-term memory

**Figure 5:**
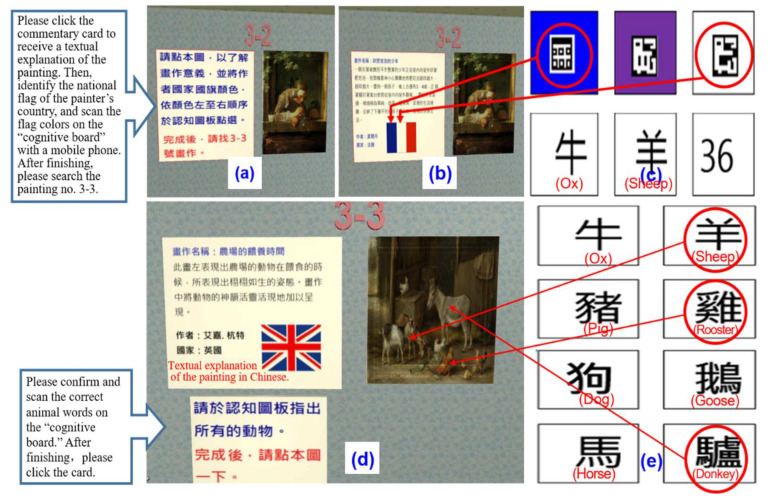
Memory test screen: (**a**) Instructions screen for the start of the short-term memory test; (**b**) Text explanation of the painting; (**c**) Confirm the color of the national flag and scan code on the cognitive board; (**d**) Instructions screen for the start of the long-term memory test; (**e**) Confirm and scan the correct animal words on the cognitive board.

In the short-term memory test, when a participant browsed the paintings, she/he not only experienced the artistic conception of the paintings but also recognized the background information and completed the short-term memory test. First, the participant entered the short-term memory testing area, and the system asked the participant to click the commentary card of the painting to receive a textual explanation, as shown in Figure 5a,b. Second, the participant was requested to move his or her eyes to the “cognitive board” to identify the national flag of the painter’s country of birth (see Figure 5c). She/he had to scan the icon codes of the flag colors on the “cognitive board” with a mobile phone for confirmation. If all the colors were answered correctly, 3 points were awarded; if only two colors were reported correctly, 2 points were awarded; 1 point was awarded for only one color correctly reported.

B. Long-term memory

The long-term memory test was mainly for evaluating semantic memory. Animals were used as the test content, as in the MoCA and MMSE. The participant was guided to recall the names of the animals in the paintings. When the participant entered the long-term memory test, she/he was asked to finish viewing the painting (see Figure 5d), and then to recall the animal’s name on the “cognitive board” and scan the animal’s code with a mobile phone to confirm (see Figure 5e). After completion, the participant was asked to recall the answers in a short-term memory test of delayed recall.

4. Vision and processing test

**Figure 6:**
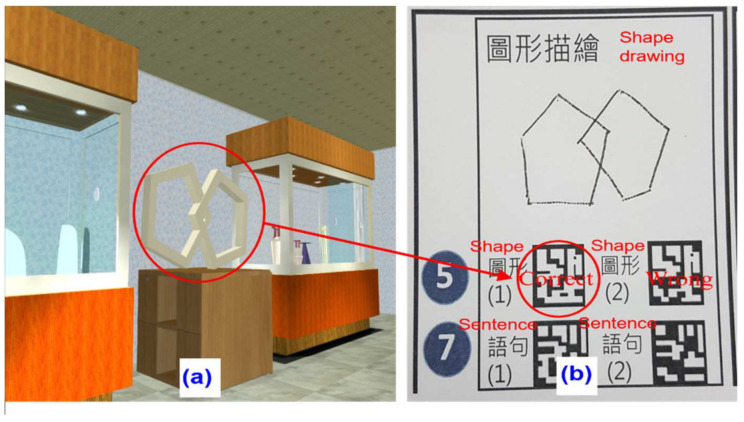
Vision and processing test screen: (**a**) A specific pattern needs to be identified; (**b**) A specific shape needs to be drawn on the cognitive board. If correct, scan the “no. 1” code with a mobile phone.

The vision and processing test was mainly for the assessment of participant judgment and ability to process visual images. There were two subtests on specific pattern identification and specific pattern drawing. Participants followed the instructions to search and identify a pattern, as shown in Figure 6a. Then, she/he was asked to draw the shape on the “cognitive board” (see Figure 6b). If the answer was correct, the tester scanned the “Shape no. (1)” code with the mobile phone, and if it was wrong, the tester scanned the “Shape no. (2)” code.

5. Attention test

**Figure 7:**
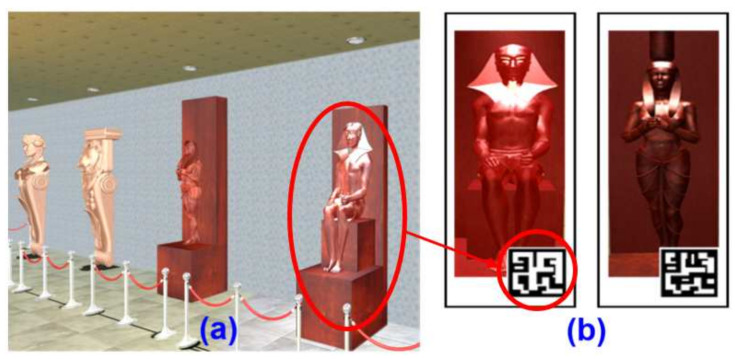
Attention test screen: (**a**) A specific cultural relic will be shown flashing; (**b**) The specific cultural relic needs to be confirmed by scanning the code on the cognitive board.

The attention test was mainly for the assessment of the participant’s attention to a special signal. During cultural relic navigation, a specific cultural relic was shown as a flashing signal, as shown in Figure 7a. When the flashing signal disappears (approximately 5 s), the participant was asked to identify the cultural relic on the “cognitive board” and scan the cultural relic code with a mobile phone for confirmation (see Figure 7b).

6. Language test

**Figure 8:**
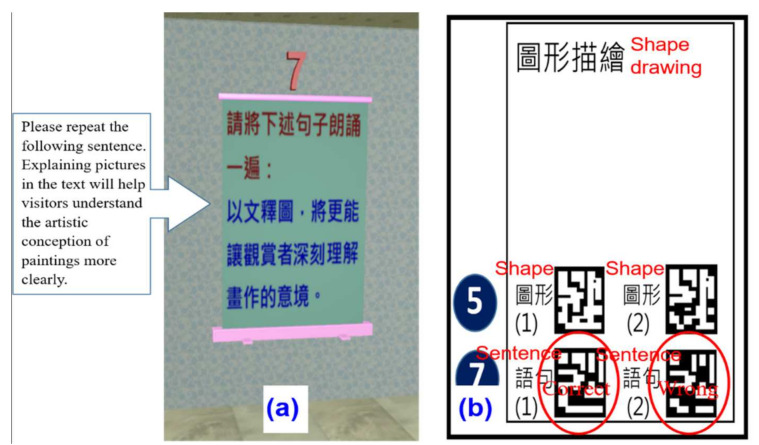
Language test screen: (**a**) Instructions screen for repeating a sentence (i.e., the blue words); (**b**) The repeated sentence needs to be confirmed by the tester. If correct, the “Sentence no. (2)” code should be scanned with the mobile phone.

The language test assessed sentence rehearsal ability. During cultural relic navigation, an instruction for repeating a sentence appeared, as shown in Figure 8a. The participant had to repeat the sentence, and the tester assisted in judging whether it was correct or not. If the answer was correct, the tester scanned the code for “Sentence no. (1)” on the “cognitive board;” if it was wrong, the tester scanned the code for “Sentence no. (2)” (see Figure 8b).

7. Oral comprehension test

**Figure 9:**
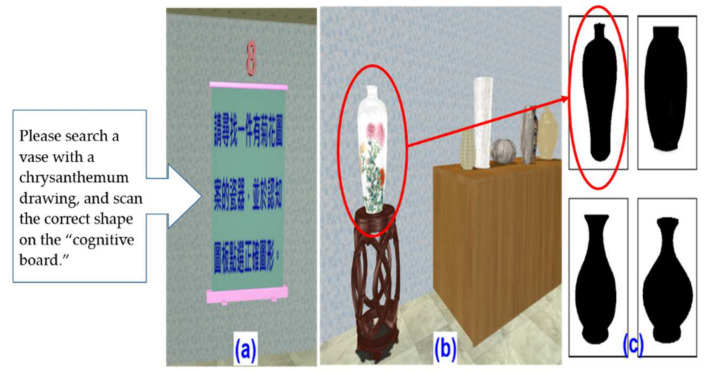
Oral comprehension test screen: (**a**) Instructions screen for searching for a specific artwork (e.g., lacquerware with chrysanthemum drawing); (**b**) The target; (**c**) Confirm and scan the correct shape on the “cognitive board”.

The oral comprehension test evaluated comprehension and action abilities. Participants were asked to search and identify artworks according to specific instructions, as shown in Figure 9a. When the participant found the designated artwork, she/he had to scan the artwork shape on the “cognitive board.” The system gave feedback on the mobile phone “做的很好” (i.e., “Great work”) whether they had scored correctly or not (see Figure 9b).

8. Calculation test

**Figure 10:**
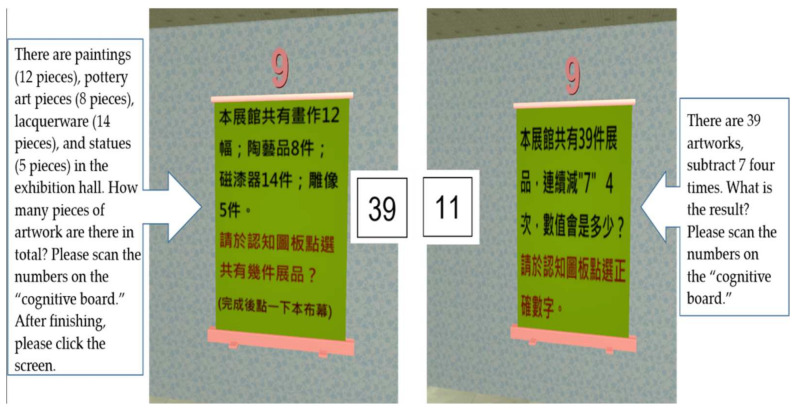
Calculation test screen: (**a**) Instructions screen for adding numbers: there are paintings (12 pieces), pottery art pieces (8 pieces), lacquerware (14 pieces), and statues (5 pieces) in the exhibition hall. How many pieces of artwork are there in total? (**b**) Instructions screen for number subtraction: there are 39 artworks, subtract 7 four times. What is the result?

The calculation test measured numerical calculation ability. After participants had completed most of the tour and returned to the lobby, there was a calculation problem shown on the presentation screen. The participants used mobile phones to scan the correct number on the cognitive board (see Figure 10).

9. Episodic memory test

**Figure 11:**
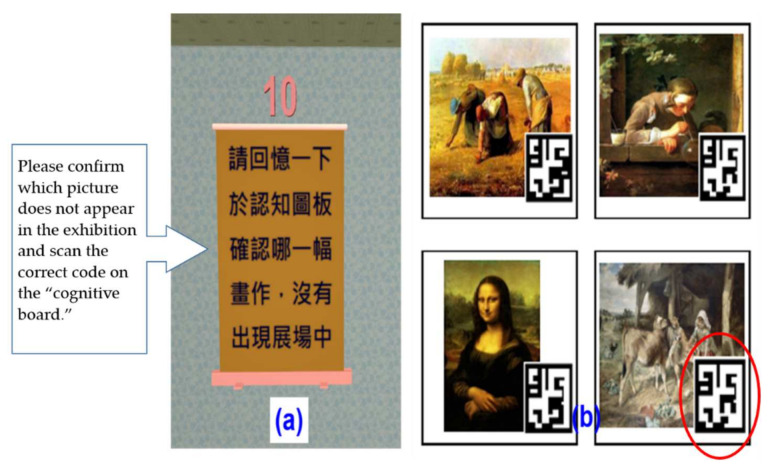
Episodic memory test screen: (**a**) Instructions screen for confirming which picture does not appear in the exhibition; (**b**) Confirm and scan the correct code on the cognitive board.

The episodic memory test was used for participants to evaluate their recall of the entire visit experience. After completing the calculation test, a memory problem was shown on the presentation screen. For example, the participant confirmed which picture did not appear in the exhibition and used a mobile phone to scan the code for the answer (see Figure 11).

10. Test results

**Figure 12:**
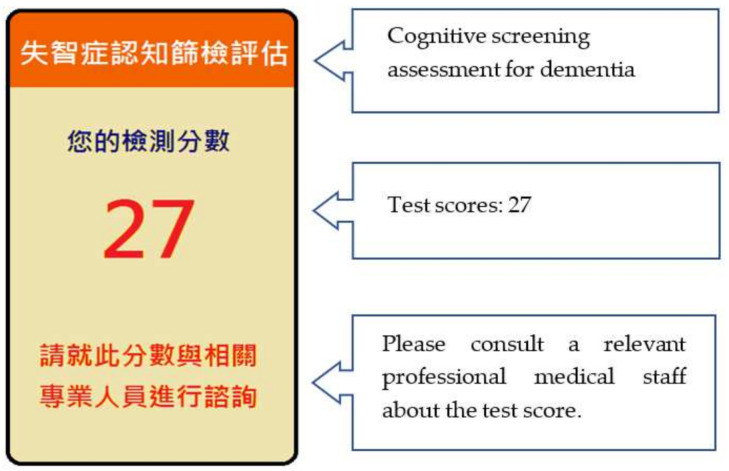
The test results screen shown on the mobile phone.

When the participant completed all the test items, the “AR recognition interface” automatically displayed the test scores, as shown in Figure 12. For the test scores, participants were encouraged to consult relevant professional medical staff to obtain accurate information about dementia.

### 2.2. Experimental Design

#### 2.2.1. Independent Variable

VSIDCS was compared with the traditional MoCA and MMSE in two aspects: One is based on the scoring results of the three tests to evaluate whether VSIDCS can meet screening objectives and be consistent with the MoCA and MMSE. Another is to explore whether VSIDCS has significantly higher involvement and emotions than the MoCA and MMSE. Therefore, the independent variable is the screening tool for initial dementia, including three levels: VSIDCS, MoCA, and MMSE.

#### 2.2.2. Dependent Variables

1. Involvement

Involvement is the enthusiasm that you feel when you care deeply about something. Previous studies found that the higher the involvement in the questionnaire-answering process is, the higher the response rate to the questionnaire is and the more opinions are provided [32]. To effectively measure a testees’ involvement in the three screening methods, the Personal Involvement Inventory (PII) questionnaire was used. The original PII was proposed by Zaichkowsky (1985) [33] and revised in 1994 (Zaichkowsky, 1994) [34]. There are 10-item measures scored on a 1 to 7 scale, including “important” (as opposed to “unimportant”); “interesting” (“boring”); “relevant” (“irrelevant”); “exciting” (“unexciting”); “means a lot to me” (“means nothing”); “appealing” (“unappealing”); “fascinating” (“mundane”); “valuable” (“worthless”); “involving” (“uninvolving”); and “needed” (“not needed”).

2. Emotions

Emotions are positive or negative experiences associated with a particular pattern of physiological activity [35]. Many items have been developed to measure emotions. In some theories, cognition is an important aspect of emotions [36]. Roseman (2001) [37] demonstrated that emotions are responses to events and are associated with a person’s goals and motivations. Éthier et al. (2006) [38] used six items to explain cognitive appraisal of a situational state (liking, joy, pride, dislike, frustration, and fear) and found that good media quality had a positive effect on liking, joy, and pride. This study uses cognitive appraisal (i.e., liking, joy, and pride) to evaluate emotions in VSIDCS, MoCA, and MMSE. In the liking aspect there are three sub-items: appreciation, liking, and preference. There are three sub-items in joy: pleasure, enjoyment, and enthusiasm. Pride also has three sub-items: self-confidence, pride, and self-praise. A nine-point Likert scale (1 = not at all, 5 = moderately, and 9 = very much) was used to measure each item.

### 2.3. Participants and Procedure

This study focused on the development and piloting of a cognitive screen/test; there were twenty-four middle-age people (F 10 and M 14 between 50 and 65 years of age and with an average age of 58.7 years) who were selected to participate in evaluation of the effects of involvement and emotions in VSIDCS, MoCA, and MMSE. All participants were fully informed and signed a consent form. They were paid a nominal fee of NTD 500 as compensation for their time.

First, before the test, participants would be taught how to operate the system, including basic operations such as forward, backward, and steering. If operations were alright, the instructor would conduct an operation demonstration and explain any precautions so that participants understood the entire operation process. Second, each participant was required to sign the “Research Participant Consent Form.” The consent form clearly stated the operating procedures and was explained by an instructor. The operating procedure was classified according to the nine items listed in Table 2, and ten tests were carried out in sequence. Additionally, each test situation in the VR cultural exhibition was also numbered for easy identification.

At the beginning of the test, participants were guided to the entrance of the VSIDCS, then operated the system themselves. During the test, if the participant had any questions, the instructor would provide assistance. As answers had to be confirmed by using the mobile phone to scan the “cognitive board,” the answers on the “cognitive board” were also categorized and numbered, as shown in Figure 2. Throughout the experiment, an instructor would assist the participant in completing each test, including drawing a specific pattern. There was no time limit for each test item, to avoid affecting the mood of the participants. VSIDCS is a passive system; if there is no action from the participant, there will be no changes on the screen. Each participant who completed the VSIDCS was asked to complete the paper test of MoCA and MMSE tests. To avoid test interference, the interval between each test had to be at least 30 min (note: there were actually 2 participants whose next test proceeded more than 1 week later, 5 participants more than 3 days, and 5 participants between 1–2 days. Only 3 participants had 30 min between each test). After completing each test, the participant needed to complete the involvement and emotion questionnaires.

## 3. Results and Discussion

### 3.1. Consistency Analysis of VSIDCS, MoCA and MMSE

Table 3 shows that there is a high degree of correlation among the three scales. The scores for VSIDCS are significantly consistent with those of the MoCA and MMSE. Therefore, it is convincing that VSIDCS can be used to screen the symptoms of dementia; the results of this test can provide the basis for the diagnosis of dementia.

Generally, the diagnosis of initial dementia is based on medical history collection and behavioral observation. Clinicians can make a diagnosis by observing the neurological and neuropsychological characteristics of patients [39]. Currently, several neuropsychological screening tests are often used to assess cognitive impairment and perform follow-up diagnoses. If there are abnormal signs, more comprehensive tests, such as computerized tomography (CT), magnetic resonance imaging (MRI), single-photon emission computed tomography (SPECT), and positron emission tomography (PET), can be performed. These advanced medical imaging techniques can effectively rule out other brain diseases, such as meningioma or subdural hematoma [40]. In addition, medical imaging can also be used to predict the transition from MCI to Alzheimer’s disease [41].

Although the diagnostic accuracy of hippocampal volumetry is very high, it is not easy to measure such in most hospitals, especially in the early stages of disease [42]. Therefore, the early stage of dementia (i.e., mild cognitive impairment) is mostly assessed through neuropsychological examinations. The MoCA and MMSE are valuable tools for detecting early cognitive decline and early dementia. Evaluation of VSIDCS reveals it to be significantly consistent with the MoCA and MMSE, so it can be appropriately used as a cognitive screening tool for initial dementia. Additionally, the average VSIDCS, MoCA, and MMSE scores were 27.1, 26.8, and 25.5, respectively. The critical scores of the VSIDCS could follow those of the MoCA; a score of 26 or more is normal.

### 3.2. The Effect of VSIDCS, MoCA, and MMSE on Involvement

Table 4 presents the variance analysis of VSIDCS, MoCA and MMSE for participant involvement. The results show that the three initial dementia evaluation methods have significant differences in involvement. To understand these differences, Tukey’s post hoc test was used for pairwise comparison of the three methods, and the results are shown in Table 5. The results show that the effect of VSIDCS on involvement is better than that of the MoCA and MMSE. Involvement is a person’s engagement with and attention given to a specific situation or thing for a period of time [43]. It is a person’s focus on an activity, which has a special feeling to him/her. The reason for higher scores of involvement in the VSIDCS may be that there is a more vivid visual and operational context during the test, and that participants can take the test with the mood of appreciating art. When participants are immersed and paying attention to the appreciation of cultural relics, they may forget they are taking a test. The MoCA and MMSE are mainly tested on paper; perhaps introducing some technical elements such as VR and AR to change traditional paper tests could make participants more willing to be involved in thinking and answering questions. The results show that there is better involvement in VSIDCS; by combining tests with thematic virtual reality (e.g., cultural exhibitions), contextual involvement can be spurred through more significant sensory input.

### 3.3. The Effect of VSIDCS, MoCA and MMSE on Emotions

Table 6 presents the variance analysis of VSIDCS, MoCA and MMSE for participant emotions. The results show that the three initial dementia evaluation methods have significant differences for emotions. To understand the differences, Tukey’s post hoc test was used for pairwise comparison of the three methods, and the results are shown in Table 7. The results show that the effect of the VSIDCS on emotions is better than that of the MoCA and MMSE. Mehrabian (1976) [44] found that emotions constitute one of the key elements of people’s psychological responses and are developed to assess whole environments and people’s responses to them. A specific environment can cause emotional reactions in an individual, which causes the individual to approach or avoid the environment. Gable (2013) [45] described that emotions might be important mediating links between social motivation and social outcomes. The total score of the emotion questionnaire used in this study was 81; a score of 40.5 was considered neutral. When the score of emotion evaluation is higher, the individual will get closer to the environment. In the results of the experiment, the average VSIDCS score was 60.4 (the maximum was 66, and the minimum was 54); the average MoCA score was 53 (the maximum was 57, and the minimum was 45); and the average MMSE score was 50.9 (the maximum was 57, and the minimum was 45). According to Murriello’s research (2015) [30], an exhibition promotes essential and relevant dialog through its collections and makes those objects meaningful to a variety of visitors. This concept was applied in the VSIDCS through introduction of VR of the exhibition, and participants’ emotions were improved through interactive dialogs. Although there are some picture and image interactions in the MoCA, such as drawing a clock and saying the name of an animal, too few questions measure this interaction. The MMSE measures even fewer such interactions, only drawing two intersecting pentagons. Our study demonstrated that test contents positively affect the cognitive appraisal of a situational state, and the more positive the evaluation of the test, the higher the intensity of emotions of liking, joy, and pride. Accordingly, it was confirmed that an interactive virtual situation applied in an initial dementia test can result in better emotions arising from appraisals of the test.

## 4. Conclusions

In aging countries, dementia has become an increasingly serious public health problem. For the evaluation of dementia symptoms, paper questionnaires are currently mainly used for testing. VSIDCS was primarily based on the standard traditional dementia test in order to change the paper tests into a computer test with VR and AR environments, performed in the same way as traditional tests. The results show that the evaluation effect of VSIDCS is significantly consistent with that of the traditional MoCA and MMSE, which means that VSIDCS can be used to screen the symptoms of dementia. In addition, this study found that VSIDCS can induce better involvement and emotion than the MoCA and MMSE. Therefore, participants will be engaged to complete the VSIDCS test and be more proactive, and then be willing to participate in, think about, and answer the questions.

MCI is a neurological disorder that mainly occurs in middle-aged individuals and older individuals. Epidemiological studies have shown that MCI may be the initial stage of dementia [15,16,46]. Both MoCA and MMSE use cognitive function assessments to assess the degree of dementia. Therefore, evaluation of MCI is also carried out by the dementia test. If the VSIDCS test score is during the initial stage of dementia, it is also considered MCI.

Due to the limitations of VR, some objects will appear relatively dull and of poor realism in the VSIDCS, but this does not affect the test results. Since VSIDCS is administered on a computer, if a person is not familiar with computer operations, she/he needs some practice before she/he can take the test. This problem must be considered. Additionally, the VSIDCS test is performed in the same way as traditional paper tests. Therefore, the VSIDCS test is best done in a medical institution, and a professional should conduct the test with the person to explain what it all means.

In general, the situational dementia cognition test constructed by visiting 3D virtual cultural relics can evaluate situational cognition as effectively as MoCA and MMSE. It is believed that VSIDCS can motivate and attract middle-aged people to participate in cognitive screening of dementia and continuous follow-up tracking. Because this study focused on the development and piloting of the cognitive screen/test, there was a small sample of middle-age people (average age is 58) that participated. In the future, an advanced study will be considered with people at risk of cognitive impairment at different ages: 50–65, 66–80, and 81+ in a cross-over design, with the order mixed up to obtain complete assessment information.

## Figures and Tables

**Table 1:** Characteristics of the VSIDCS.

Items	Characteristics
1. Function	1. Assessment of cognitive decline in initial dementia
2. Objects	1. Middle-aged people (over 50 years old)
3. Reference screening tools	1. Montreal Cognitive Assessment Test (MoCA) 2. Mini-Mental State Examination (MMSE)
4. Theoretical basis	1. Episodic memory 2. Cognitive impairment
5. Design concept	1. Design ideas Dementia screening is constructed as a detection scenario consisting of an intellectual journey through a virtual cultural tour. 2. Application concept Encouraging and attracting middle-aged people to have a higher willingness to participate in cognitive screening and to continue to follow up.
6. Situational design	1. Context Participants visit 4 major cultural relic exhibitions (paintings, pottery, lacquerware, and statues) for guided tours to view and interact with artworks. 2. Situational purpose During the cultural tour, through virtual reality operation and augmented reality assessment, participants will complete orientation, short-term memory, long-term memory, vision and processing, attention, language, oral comprehension, calculation, episodic memory, and control evaluation.
7. Behavioral response	1. Orientation, short-term memory, long-term memory, vision and processing, attention, language, oral comprehension, calculation, and episodic memory.
8. Evaluation process	1. Participants visit 4 major cultural relic exhibitions according to set procedures. 2. When the participant completes item operations, question answers, and action responses during the visit, the test scores are evaluated according to the response results.

**Table 2:** Evaluation items, methods, and scoring of VSIDCS.

Categories	MoCA	MMSE	VSIDCS
1. Orientation	Date: Day, Month, Year; Place	Date: Day, Month, Year, Season; Place	Date: Month, Year; Find a specific place
	Points: 6	Points: 10	Points: 6
2. Short-term memory	Read the list of words; Delay recall	Delay recall	Identify the national flag; Delay recall
	Points: 5	Points: 3	Points: 5
3. Long-term memory	Names of animals	None	Names of animals in a painting
	Points: 3	Points: -	Points: 3
4. vision and processing	Sequential connection; Copy cube; Draw CLOCK	Draw two intersecting pentagons	Identify a specific pattern; Draw a specific pattern
	Points: 5	Points: 1	Points: 4
5. Attention	Read the list of digits and letters	Name three unrelated objects; Recall	Identify a specific artwork
	Points: 3	Points: 3	Points: 3
6. Language	Repeat a sentence; Name maximum number of words that begin with a letter	Name a specific object; Recall; Follow an instruction; Write a sentence	Repeat a sentence; Recall
	Points: 3	Points: 5	Points: 3
7. Readability	Similarity between	Read the following and do what it says	Read the following and search for specific artworks
	Points: 2	Points: 3	Points: 2
8. Calculation	Serial 7 subtraction starting at 100	Serial 7 subtraction starting at 100	Calculate number of art exhibitions; Serial 7 subtraction starting from the total number of art exhibitions
	Points: 3	Points: 5	Points: 3
9. Episodic memory	None	None	Identify the painting visited
	Points:-	Points:-	Points: 1
Total	Points: 30	Points: 30	Points: 30

MoCA: Montreal Cognitive Assessment; MMSE: Mini-Mental State Examination; VSIDCS: Virtual scenario Initial Dementia Cognitive Screening.

**Table 3:** Correlation analysis of VSIDCS, MoCA, and MMSE scores.

		VSIDCS	MoCA	MMSE
VSIDCS	Pearson correlation	1	0.774 **	0.795 **
	Sig. (two-tailed)		0.000	0.000
	N	24	24	24
MoCA	Pearson correlation	0.774 **	1	0.810 **
	Sig. (two-tailed)	0.000		0.000
	N	24	24	24
MMSE	Pearson correlation	0.795 **	0.810 **	1
	Sig. (two-tailed)	0.000	0.000	
	N	24	24	24

** Correlation is significant at level 0.01 (two-tailed). VSIDCS: Virtual scenario Initial Dementia Cognitive Screening; MoCA: Montreal Cognitive Assessment; MMSE: Mini-Mental State Examination.

**Table 4:** One-way ANOVA (analysis of variance) for the effects of VSIDCS, MoCA, and MMSE on involvement.

Source of Variation	*SS*	*df*	*MS*	*F*	*p-*Value
Between-Group	1977.444	2	988.722	120.179	0.000
Within-Group	567.667	69	8.22705		
Total	2545.111	71			

**Table 5:** Tukey’s post hoc tests for the effects of VSIDCS, MoCA, and MMSE on involvement.

(I) Display Types	(J) Display Types	Mean Difference (I–J)	Std. Error	*p* Value
VSIDCS	MoCA	9.250 *	0.828	0.000
	MMSE	12.333 *	0.828	0.000
MoCA	VSIDCS	−9.250 *	0.828	0.000
	MMSE	3.083 *	0.828	0.001
MMSE	VSIDCS	−12.333 *	0.828	0.000
	MoCA	−3.083 *	0.828	0.001

Notes: * Significant at 0.05 level.

**Table 6:** One-way ANOVA (analysis of variance) for the effects of VSIDCS, MoCA, and MMSE on emotions.

Source of Variation	*SS*	*df*	*MS*	*F*	*p*-Value
Between-Group	1193.250	2	596.625	57.961	0.000
Within-Group	710.250	69	10.293		
Total	1903.500	71			

**Table 7:** Tukey’s post hoc tests for the effects of VSIDCS, MoCA, and MMSE on emotions.

(I) Display Types	(J) Display Types	Mean Difference (I–J)	Std. Error	*p* Value
VSIDCS	MoCA	7.375 *	0.926	0.000
	MMSE	9.500 *	0.926	0.000
MoCA	VSIDCS	−7.375 *	0.926	0.000
	MMSE	2.125	0.926	0.063
MMSE	VSIDCS	−9.500 *	0.926	0.000
	MoCA	−2.125	0.926	0.063

Notes: * Significant at 0.05. VSIDCS: Virtual scenario Initial Dementia Cognitive Screening; MoCA: Montreal Cognitive Assessment; MMSE: Mini-Mental State Examination.

## Data Availability

The data presented in this study are available on request from the corresponding author. The data are not publicly available due to privacy reasons.
